# Efficacy of Co-administration of Liuwei Dihuang Pills and Ginkgo Biloba Tablets on Albuminuria in Type 2 Diabetes: A 24-Month, Multicenter, Double-Blind, Placebo-Controlled, Randomized Clinical Trial

**DOI:** 10.3389/fendo.2019.00100

**Published:** 2019-02-22

**Authors:** Ruifeng Shi, Yanping Wang, Xiaofei An, Jianhua Ma, Tongzhi Wu, Xiaojin Yu, Su Liu, Liji Huang, Lijuan Wang, Jingshun Liu, Jing Ge, Shanhu Qiu, Han Yin, Xiaolai Wang, Yao Wang, Bingquan Yang, Jiangyi Yu, Zilin Sun

**Affiliations:** ^1^Department of Endocrinology, Zhongda Hospital, School of Medicine, Institute of Diabetes, Southeast University, Nanjing, China; ^2^Department of Endocrinology, Wuxi Third People's Hospital, Wuxi, China; ^3^Department of Endocrinology, Jiangsu Province Hospital of Traditional Chinese Medicine, Nanjing, China; ^4^Department of Endocrinology, Nanjing First Hospital, Nanjing Medical University, Nanjing, China; ^5^Discipline of Medicine and Centre of Research Excellence in Translating Nutritional Science to Good Health, University of Adelaide, Adelaide, SA, Australia; ^6^Department of Epidemiology and Biostatistics, School of Public Health, Southeast University, Nanjing, China

**Keywords:** diabetic nephropathy, urinary albumin, creatinine ratio, Liuwei Dihuang, Ginkgo biloba

## Abstract

**Purpose:** We investigated the effects of Traditional Chinese Medicine (TCM) on the occurrence and progression of albuminuria in patients with type 2 diabetes.

**Methods:** In this randomized, double-blind, multicenter, controlled trial, we enrolled 600 type 2 diabetes without diabetic nephropathy (DN) or with early-stage DN. Patients were randomly assigned (1:1) to receive Liuwei Dihuang Pills (LWDH) (1.5 g daily) and Ginkgo biloba Tablets (24 mg daily) orally or matching placebos for 24 months. The primary endpoint was the change in urinary albumin/creatinine ratio (UACR) from baseline to 24 months.

**Results:** There were 431 patients having UACR data at baseline and 24 months following-up in both groups. Changes of UACR from baseline to follow-up were not affected in both groups: −1.61(−10.24, 7.17) mg/g in the TCM group and −0.73(−7.47, 6.75) mg/g in the control group. For patients with UACR ≥30 mg/g at baseline, LWDH and Ginkgo biloba significantly reduced the UACR value at 24 months [46.21(34.96, 58.96) vs. 20.78(9.62, 38.85), *P* < 0.05]. Moreover, the change of UACR from baseline to follow-up in the TCM group was significant higher than that in the control group [−25.50(−42.30, −9.56] vs. −20.61(−36.79, 4.31), *P* < 0.05].

**Conclusion:** LWDH and Ginkgo biloba may attenuate deterioration of albuminuria in type 2 diabetes patients. These results suggest that TCM is a promising option of renoprotective agents for early stage of DN.

**Trial registration:** The study was registered in the Chinese Clinical Trial Registry. (no. ChiCTR-TRC-07000037, chictr.org)

## Introduction

Diabetic nephropathy (DN) is one of the most common causes of end-stage renal disease (ESRD) world widely, accounting for considerable morbidity and mortality in patients with both type 1 and type 2 diabetes mellitus (DM) (1, 2). Estimated GFR and albuminuria are independent risk factors which associate with progression to ESRD strongly (3). The common pathologic feature of early DN is the presence of glomerular hypertrophy, with mesangial expansion and glomerular basement membrane thickening, which cause excessive glomerular filtration and increased urinary albumin excretion. Emerging evidence shows that both renal impairment and cardiovascular events occur in patients with developed micro-albuminuria earlier for diabetes, suggesting fundamental importance to the management of microalbuminuria.

The progression of albuminuria is associated with glomerular hypertension, inflammation, and oxidative stress. The present management for microalbuminuria includes strict control of blood glucose and blood pressure, with the ACE inhibitors (ACEI) or angiotensin receptor blockers (ARB). These therapies, however, are frequently unsatisfactory, probably reflecting inadequate targeting on the pathophysiology (4). For example, the use of ACEI/ARB failed to stop the progression of nephropathy to ESRD in type 2 patients (5). The albuminuria was demonstrated to have positive correlation with renal risk in patients with type 2 diabetic nephropathy (DN) (6). Albuminuria also has been demonstrated a direct toxic effect on renal tissue, leading to progressive renal damage (7). Previous clinical trial shows that initial reduction of albuminuria can reduce the risk of ESRD (8).

In China, Traditional Chinese Medicine (TCM) has been widely used as complementary therapy for DN, including Lumbrokinase (9), Xue Shuan Tong (10), Berberine (11), huangkui (12), Salvia miltiorrhiza (13), Liuwei Dihuang (LWDH), and Ginkgo biloba. The latter two have been proposed for the prevention and treatment of DN in China and other Asian regions recently (14–17). LWDH is a classic TCM, which is reported to ameliorate oxidation and improve insulin sensitivity as well as glucose tolerance (18, 19). Ginkgo biloba contains ~24% flavone glycosides (primarily quercetin, kaempferol, and isorhamnetin) and 6% terpene lactones in weight, with a long history in the use for a variety range of conditions in China. Administration of Ginkgo biloba is associated with blood vessel dilation, reduction in blood viscosity and the density of oxygen free radicals, inhibition of platelet activating factor (20). Therefore, Ginkgo biloba may have renal protective effects in DN. In both animal models and patients with DN, Ginkgo biloba was shown to inhibit NF-κB, oxidation and accumulation of extracellular matrix (ECM) (21–23). However, whether combination of LWDH and Ginkgo biloba can prevent the progression of renal injury is unclear. This study was designed to evaluate co-administration of LWDH Pills and Ginkgo biloba Tablets on renoprotection for type 2 diabetes with normal urinary albumin excretion or microalbuminuria.

## Materials and Methods

### Subjects

This is a multicenter, randomized, double-blind, placebo-controlled trial. Briefly, patients were included if they had type 2 diabetes, urine albumin excretion (UAE) < 300 mg/d, were aged 30–70 years, provided written informed consent. Patients were excluded from participation for any of the following: (1) poor control of hypertension(>160/110 mmHg), (2) diagnosed macrovascular complications (such as myocardial infarction, stroke, transient ischemic attack, peripheral vascular disease), polyneuropathy, or proliferative retinopathy, (3) using ACEI, ARB, or statins within 6 months prior to the enrollment (in order to avoid potential effects on albumin excretion). All subjects gave written informed consent. The protocol was approved by the Human Research Ethics Committee of each study center, and was conducted in accordance with the principles of the Declaration of Helsinki as revised in 2000.

**Figure 1:**
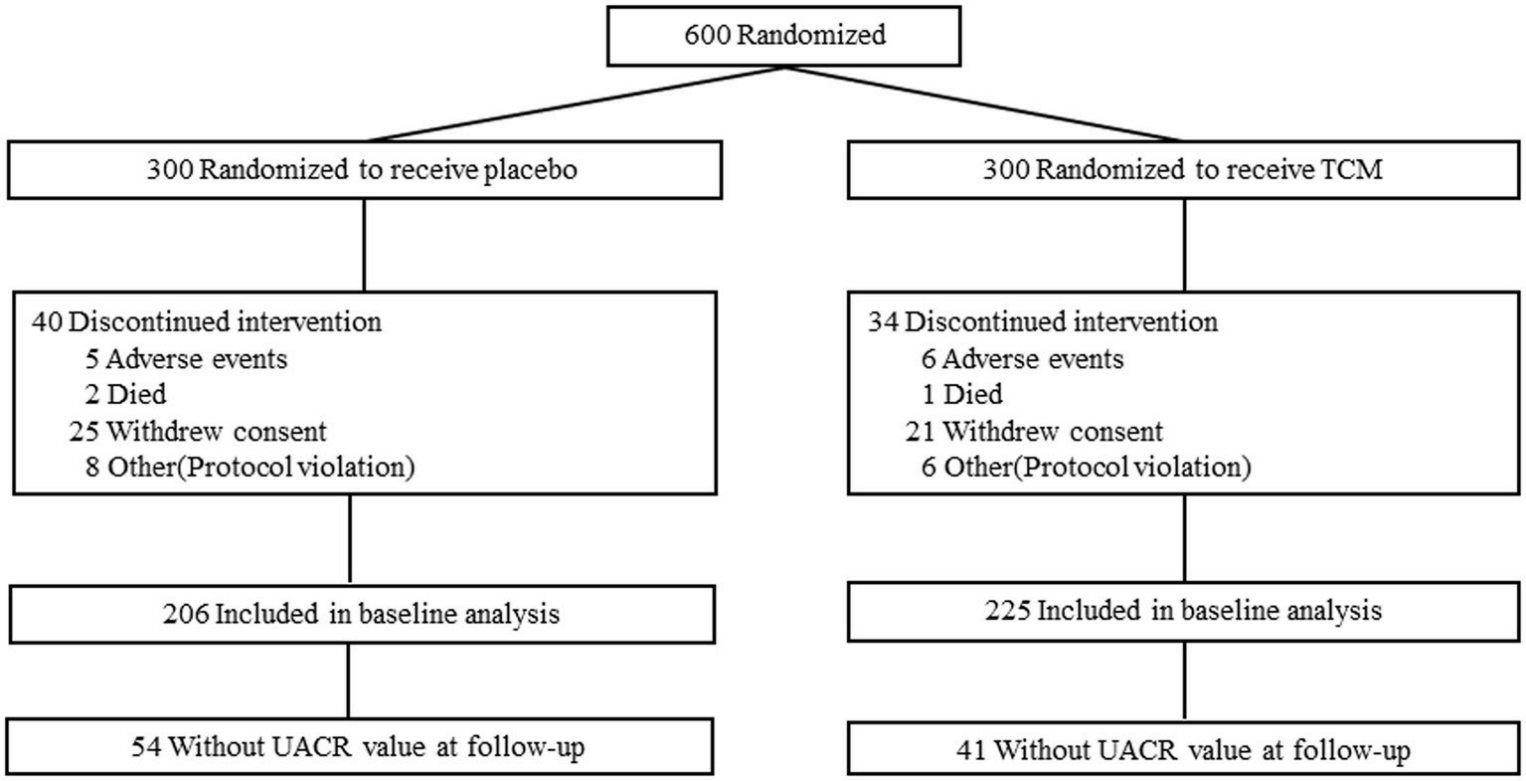
Flow of participants in the trial.

This clinical trial was compliant with the Consolidated Standards of Reporting Trials (CONSORT). Subjects were recruited and screened at 10 clinical diabetes centers according to the criteria above. After enrollment, subjects were randomized at 1:1 to receive 1.5 g LWDH Pills (8 pills/time; batch number Z41022128; Wanxi Pharmaceutical Co., Ltd.) and 24 mg Ginkgo biloba Tablets (2 tablets/time; batch number Z20027949; Yangtze River Pharmaceutical Co., Ltd.) (TCM group, *n* = 300) orally three times per day, or matching placebos (placebo group, *n* = 300) (Figure 1). Randomization was performed by an independent doctor in each clinical center with block randomization method. Patients, investigators, and the sponsor's clinical team were all blinded to treatment allocation. Subjects were followed up with clinic consultation for 2 years.

### Primary End Point

The primary outcome variable was the change in urinary albumin/creatinine ratio (UACR) before and after treatment. On the first visit, each subject was fasted overnight (at least 8 h), and attended the clinical center at 08:00. An overnight first-void urine sample was collected from each patient to measure the UACR. Normal albuminuria was defined as an UACR < 30 mg/g. Patients were considered to have microalbuminuria if their UACR ranged in 30–299 mg/g. Macro albuminuria was defined as UACR ≥300 mg/g.

### Other Outcomes

All blood samples were immediately obtained at 08:00 after overnight. Enzyme-linked immunosorbent assay was used to detect the high-sensitivity C-reactive protein (HS-CRP) (Lot 78034031, Bender Med Systems GmbH, Austria; minimum detection limit: 3 pg/ml; intra-assay CVs: 6.9%; inter-assay CVs: 13.1%), matrix metalloproteinase 2 (MMP2) (Lot 303216, R&D, USA; minimum detection limit: 0.047 ng/ml; intra-assay CVs: 5.6%; inter-assay CVs: 7.4%), soluble advanced glycation end products (sRAGE) (Lot 303510, R&D, USA; minimum detection limit: 4.12 pg/ml; intra-assay CVs: 5.7%; inter-assay CVs: 7.7%), and fractalkine (Lot 301156, R&D, USA; minimum detection limit: 0.018 ng/ml; intra-assay CVs: 2.6%; inter-assay CVs: 6.6%). The concentrations of serum AGE-peptides (AGE-P) were measured by flow injection assay (FIA) (24).

The subject was consumed a standardized breakfast (100 g steamed bread). Venous blood were sampled before and after breakfast, and fasting blood glucose (FBG), HbA1c, total cholesterol (TC), total triglyceride (TG), HDL, LDL, and postprandial blood glucose (PBG) were measured. The glomerular filtration rate (GFR) was estimated using the equation recommended by the National Kidney Foundation in the Modified Diet in Renal Disease (25).

### Adverse Events

Adverse events included cancer, stroke, coronary artery disease, bleeding, and many transient minor complaints, such as dizziness, nausea, hypoglycemia, skin itching or headache. Participants were count only for once.

### Statistical Analysis

Paired sample *T*-test is used for intragroup comparisons for clinical characteristics between the baseline and the end of follow-up. Independent samples *T*-test is used between the placebo and TCM group if the normal distribution is satisfied, otherwise, the non-parametric test is used. Family history of type 2 diabetes, history of retinopathy and the therapies between the two groups were compared using chi-squared test. Subgroup analysis was performed according to the baseline UACR (≥30 or < 30 mg/g). A probability of *p* < 0.05 was considered to be statistically significant. All analyses were performed using SPSS software (version 17.0; SPSS Inc). Data were presented as means ± SD or Median (lower quartile, upper quartile).

## Results

### Basic Characteristics

Six hundred type 2 patients were enrolled, 74 of which were lost during the follow-up, i.e., 34 patients in the TCM group and 40 in the placebo group, and the reasons for these dropouts were reported in Figure 1. There was no difference in age, gender, duration of known diabetes, BMI, blood glucose, HbA1c, SBP, DBP, HDL, LDL, TC, TG, GFR, or the presence of microalbuminuria between the two groups at the baseline. Baseline clinical characteristics were well-balanced between the two groups during the 24 months treatment (Table 1).

**Table 1:** The characteristics at baseline and 24 months treatment of placebo and TCM.

	**Placebo group**			**TCMgroup**		
	**baseline**	**end of follow-up**	***d***	**baseline**	**end of follow-up**	***d***
FBG (mmol/L)	7.45 ± 2.21	6.93 ± 1.58*	−0.51 ± 2.46	7.49 ± 2.31	6.85 ± 1.47*	−0.58 ± 2.31
PBG (mmol/L)	10.76 ± 3.83	9.73 ± 2.70*	−1.03 ± 4.37	11.00 ± 3.74	9.80 ± 2.98*	−1.13 ± 3.88
HbA1c (%)	7.28 ± 1.90	6.53 ± 1.21*	−0.66 ± 1.89	7.26 ± 1.81	6.66 ± 1.18*	−0.50 ± 1.79
SBP (mmHg)	125.62 ± 13.68	123.75 ± 11.28	−0.89 ± 14.38	125.87 ± 13.42	123.54 ± 11.67*	−2.21 ± 14.30
DBP (mmHg)	77.38 ± 8.99	75.48 ± 7.22*	−1.09 ± 8.31	77.81 ± 9.30	74.76 ± 7.37*	−2.03 ± 9.07
TC (mmol/L)	4.74 ± 0.97	4.73 ± 0.94	−0.11 ± 0.92	4.84 ± 0.90	4.94 ± 0.94*	−0.21 ± 0.91
TG (mmol/L)	1.54 ± 1.03	1.37 ± 0.83	−0.15 ± 1.29	1.56 ± 1.00	1.45 ± 1.19	0.05 ± 1.45
HDL (mmol/L)	1.26 ± 0.32	1.19 ± 0.28	−0.04 ± 0.34	1.29 ± 0.34	1.27 ± 0.37	−0.01 ± 0.26
LDL (mmol/L)	2.78 ± 0.71	2.74 ± 0.70	−0.00 ± 0.71	2.87 ± 0.78	2.76 ± 0.80	−0.08 ± 0.84
BMI (kg/m^2^)	23.99 ± 2.73	24.33 ± 2.80	−1.51 ± 6.16	23.82 ± 2.76	23.98 ± 3.09*	−0.38 ± 4.03^†^
Age (years)	60.45 ± 6.19			60.81 ± 6.36		
Male sex, *n* (%)	151 (50.33)			146 (48.67)		
Diabetes duration (years)	5.30 ± 4.51			5.65 ± 5.15		
GFR (mL/min/1.73m^2^)	86.52 ± 19.57			88.21 ± 19.98		
ACR>30 mg/g, *n* (%)	45 (17.31)			32 (12.03)		
Family history of T2DM, *n* (%)	105 (35)			109 (36.30)		
History of retinopathy disease, *n* (%)	61 (20.33)			68 (22.67)		
**GLUCOSE-LOWERING THERAPIES**, ***N*** **(%)**						
Diet only	40 (13.33)			38 (12.67)		
Sulfonylurea	120 (40)			102 (34)		
Alpha-glucosid ase inhibitor	46 (15.33)			41 (13.67)		
Glinides	18 (6)			26 (8.67)		
Insulin, insulin analog	80 (26.67)			83 (27.67)		
Metformin	115 (38.33)			104 (34.67)		
Thiazolidinedione	15 (5)			17 (5.67)		
**ANTIHYPERTENSIVE THERAPIES**, ***N*** **(%)**						
Calcium channel blockers	73 (24.33)			64 (21.33)		
Beta-blocker	15 (5)			10 (3.33)		
Diuretics	12 (4)			24 (8)		
Alpha-blocker	1 (0.33)			0		
Hydrochlorothiazide and irbesartan	0			1 (0.33)		
Vasodilator	3 (1)			2 (0.67)		

*FBG, fasting blood glucose; PBG, postprandialblood glucose; SBP, systolic blood pressure; DBP, diastolic blood pressure; TC, total cholesterol; TG, triglyceride; ACR, albumin/creatinine ratio*.

*d-value: difference between baseline and 24 months follow-up*.

Paired sample T-test was used for intragroup comparisons between the baseline and the end of follow-up, *p < 0.05.

*Independent samples T-test was used to determine the d-value between the placebo and TCM group, p < 0.05*.

*Independent samples T-test was used to determine the statistical significance between the placebo and TCM group at baseline and follow-up, all the p values > 0.05*.

*Data are presented as means ± SD*.

### Primary End Point

For all these subjects, 54 in the control group and 41 in the TCM group did not have UACR value at 24 months follow-up. There were no significant differences in UACR between the placebo (*n* = 206) and TCM group (*n* = 225) before and after treatment. The change of UACR in both groups was not affected. Patients were divided into subgroups according to the baseline values of UACR: UACR < 30 mg/g, UACR ≥30 mg/g. For patients with UACR ≥30 mg/g at baseline, the reduction of UACR value between baseline and follow-up was much more obvious in the TCM group compared with that in the placebo group [−25.50(−42.30, −9.56) vs. −20.61(−36.79, 4.31), *P* < 0.05]. Moreover, UACR decreased significantly at 24 months compared with baseline in both placebo [44.79 (35.12, 65.11) vs. 32.19(12.32, 64.99), *P* < 0.05] and TCM groups [46.21(34.96, 58.96) vs. 20.78(9.62, 38.85), *P* < 0.05] (Table 2).

**Table 2:** The effect of 24 months treatment on ACR in placebo and TCM group.

	**Placebo (*****n*** **= 206)**			**TCM (*****n*** **= 225)**		
**(mg/g)**	**Baseline**	**24 months**	***d*-value**	**Baseline**	**24 months**	***d*-value**
Total	14.32 (6.68, 23.73)	11.97 (7.49, 23.01)	−0.73(−7.47, 6.75)	15.31 (7.46, 25.37)	12.55 (7.57, 22.26)	−1.61 (−10.24, 7.17)
UACR ≥30	44.79 (35.12, 65.11)	32.19 (12.32, 64.99)*	−20.61(−36.79,4.31)	46.21 (34.96, 58.96)	20.78 (9.62,38.85)*	−25.50 (−42.30, −9.56)^†^
UACR < 30	12.63 (6.18, 18.40)	10.91 (6.69, 18.52)	−0.28 (−5.25,6.76)	13.10 (6.77, 19.23)	12.16 (7.48, 19.81)	0.96 (−6.50, 8.85)

*Total: all the subjects. Subjects were divided into tertiles: UACR < 30 mg/g, UACR ≥ 30 mg/g. d-value: difference between baseline and end of follow-up*.

Two-related-samples nonparametric tests was used for intragroup comparisons between the baseline and the end of follow-up, *p < 0.05.

*Analysis covariance was used for d-value of samples between the placebo and TCM group, p < 0.05*.

*Two-independent-samples nonparametric tests was used to determine the statistical significance between the placebo and TCM group in the baseline and end of follow-up, all the p > 0.05*.

*Data are presented as median (lower quartile, upper quartile)*.

### Onset and Remission Rate of Microalbuminuria and Macroalbuminuria

There was no significant difference in the occurrence of microalbuminuria at follow-up between the two groups for those with baseline normoalbuminuria (*P* > 0.05). Despite of the control of blood glucose, lipids and pressure, 2 patients progressed into macroalbuminuria in the placebo group (1.14%), without any occurrence among the patients treated with TCM. There was no significant reduction in the remission rate of microalbuminuria after 24 months in TCM treatment compared with control group in patients with existing microalbuminuria at baseline (65.9% vs. 48.4%, *p* > 0.05).

### UACR and Markers of Inflammation in One Center

The characteristic at the baseline between the two groups were balanced (Supplemental Table 1). No significant difference of UACR was observed between placebo and TCM groups at baseline or after intervention. Serum fractalkine (0.56 ± 0.25 vs. 0.78 ± 0.43 ng/mL, *p* = 0.01) and AGE-P (14.92 ± 2.43 vs. 19.29 ± 4.96 μg/mL, *p* = 0.03) concentrations were lower, whereas sRAGE concentrations (2,242.75 ± 359.67 vs. 1,832.63 ± 324.83 pg/mL, *p* = 0.04) in the TCM group were higher than those in placebo at 24 months. However, there was no significant difference in CRP (0.73 ± 0.59 vs. 0.96 ± 0.79 mg/L, *p* > 0.05) or MMP2 (186.66 ± 72.68 vs. 173.43 ± 59.91 ng/mL, *p* > 0.05).

### Adverse Events

The overall occurrence of adverse events was similar between the two groups. A total of 53 adverse events were reported (26 in the placebo group and 27 in the TCM group). Serious adverse events were: coronary artery disease (2 patients in both groups), cerebrovascular accident (4 patients in TCM group and 5 patients in control group), bleeding (1 patient in control group) and cancer (4 patients in TCM group and 2 patients in control group).

## Discussion

In this multicenter, randomized, double-blind, and placebo-controlled prospective trial, we find that co-administration of 1.5 g LWDH and 24 mg Ginkgo biloba three times per day for 24 months is benefit to type 2 diabetes for microalbuminuria (as reflected by measures in UACR), associated with downregulation of inflammatory markers (i.e., fractalkine, AGE-P). The combined therapy may ameliorate the microalbuminuria in the patients with early DN. These observations provide evidence that Ginkgo biloba and LWDH are attractive alternatives for the management of DN.

Both Ginkgo biloba and LWDH are commonly prescribed for a variety of clinical conditions, without significant side effects. Increasing interest has been placed on their efficacy and safety for the management of early DN (16, 26–28) in China. However, the strength of these studies was limited by small sample size, short-duration, and lack of appropriate control. The present study represents the first large-scale, multicenter, randomized, double-blind, placebo-controlled trial, which evaluates the effects of combined treatment with LWDH and Ginkgo biloba on the progression of DN among relatively well-controlled type 2 diabetes for 24 months. We did not observe a significant difference in the change of UACR before and after intervention, but the change of UACR was alleviated significantly for a subgroup of patient with microalbuminuria. We failed to reveal statistical difference in UACR between the two groups probably due to the fact that patients with microalbuminuria were only a small portion of all these subjects (18.22% in TCM group and 15.05% in placebo group). It seems that this treatment have an effect on patients with microalbuminuria but not on those with normal albuminuria. TCM treatment didn't change the UACR for patients without albuminuria while they accounted for a large part of the study. Therefore, addition of TCM to standard clinical intervention appears to be promising for the DN with microalbuminuria. A larger clinical trial for this population is needed to confirm this conclusion.

As previously reported, diabetes mellitus is associated with multiple inflammatory responses, including AGEs accumulation, leukocyte infiltration, ECM depositing, cytokines, and adhesion molecule expression, which may contribute to the development of renal impairment (29, 30). In the present study, we demonstrated positive relationships of AGE-P and fractalkine, and a reverse relationship with sRAGE after 24 months intervention. In the circulation, small-sized AGE-P may act as a reactive intermediate to cause tissue injury via binding to susceptible target proteins both within and outside the vasculature and hence forming “second-generation” AGEs. We have reported that AGE-P represents a valuable marker for predicting the severity DN, and AGEs upregulates fractalkine expression in human renal mesangial cells (HRMC) (31). Fractalkine, an important chemoattractant with adhesiveness functions, acts to induce macrophage recruitment that may cause abnormality of the ECM metabolism and consequent deposition of excessive ECM (32). In contrast, sRAGE has been described as a “sponge” for AGEs which counteracts the detrimental effects of cellular RAGE by binding with serum AGEs and AGE-P (33). It has been shown that the lower level of sRAGE is associated with the higher risks of diabetes, coronary heart disease, and all-cause mortality (34).

Some studies contribute the protection of TCM to their anti-oxidative and anti-inflammatory properties. For example, LWDH was demonstrated to decrease cytokinesin mice with experimental autoimmune encephalomyelitis (35). Ginkgo biloba was reported to improve platelet function, to alter platelet-vessel wall interactions, and to reduce malondialdehyde levels in platelets in type 2 patients (36). In addition, Gingko biloba was also shown to reduce relative total superoxide dismutase activity in patients with DN (37). In the present study, TCM treatment leads to a lower fractalkine level and a higher sRAGE level.

Because of reducing the ability of the blood to clot, Gingko biloba was reported to increase the risk of bleeding with high dose varying from 120 to 240 mg daily (38). No one reported bleeding with lower dose (between 35 and 70 mg daily). The safety of Gingko biloba extract for early DN still needs further research to estimate if there is any side effect. Previous study and our trial did not observe the related side effect. So, patients with bleeding related disease should be treated with cautions.

Our study has several limitations. Firstly, a 24 months follow-up was relatively short to evaluate the natural progression of DN, and the prevention of TCM on the DN. Secondly, there was a relatively high rate of premature withdrawals due to the longer follow-up period. Thirdly, this is a phase II trial and there was no formal sample size calculation due to the absence of available research on this topic at that time. As a result, the negative result in the primary endpoint may be subject to false-negative error. Finally, we were unable to obtain blood samples from all centers to measure inflammatory markers.

In summary, this multicenter, randomized, double-blind, placebo-controlled trial suggests that LWDH and Ginkgo biloba may be effective for managing DN. Further research to investigate the renal effects of co-administration prospectively is underway.

## Author Contributions

RS analyzed part of the data and wrote the manuscript. YanW collected the data and analyzed part of the data. ZS and JY involved in the design/implementation of the overall study, designed the analysis plan, and supervised the analysis and manuscript. TW and SQ contributed to the discussion and reviewed the manuscript. XY performed the statistical analyses guidance. XA, JM, SL, LH, LW, JL, JG, HY, XW, YaoW, and BY involved in the implementation of the overall study.

### Conflict of Interest Statement

The authors declare that the research was conducted in the absence of any commercial or financial relationships that could be construed as a potential conflict of interest.

## Abbreviations

TCM: Traditional Chinese Medicine.
