# Supplementary material for: Efficacy of Co-administration of Liuwei Dihuang Pills and Ginkgo Biloba Tablets on Albuminuria in Type 2 Diabetes: A 24-Month, Multicenter, Double-Blind, Placebo-Controlled, Randomized Clinical Trial
Source: Front Endocrinol (Lausanne). 2019 Feb 22;10:100. doi: 10.3389/fendo.2019.00100 (PMC6402447; doi:10.3389/fendo.2019.00100)
Supplement: Supplementary file 1 [file Table_1.docx]

Supplemental Table 1. The baseline characteristics in one center.

|  | Placebo | TCM | |
| --- | --- | --- | --- |
| Age (years) | 59+6.01 | | 62.68+5.53 |
| Male sex, n (%) | 14 (31.11) | | 24 (55.81) |
| Diabetes duration (years) | 5.26+4.16 | | 6.04+5.27 |
| TC(mmol/L) | 4.44+0.97 | | 5.02+0.95 |
| TG(mmol/L) | 1.12+0.79 | | 1.19+0.56 |
| HDL(mmol/L) | 1.29+0.30 | | 1.32+0.32 |
| LDL(mmol/L) | 2.63+0.66 | | 3.12+0.72 |
| HbA1c(%) | 7.10+1.81 | | 7.20+1.13 |
| ACR (mg/g) | 11.14 (6.63,16.72) | | 13.2 (8.36, 22.13) |

Paired Student’s t-test was used for intragroup comparisons between the baseline and the end of follow-up. *: *p* < 0.05.

Unpaired Student’s t-test was used to determine the d-value between the placebo and TCM group. †: *p* < 0.05.

Data are presented as means ± SD or Median (lower quartile, upper quartile)
